# Clinical Relevance of the First Domomedicine Platform Securing Multidrug Chronotherapy Delivery in Metastatic Cancer Patients at Home: The inCASA European Project

**DOI:** 10.2196/jmir.6303

**Published:** 2016-11-25

**Authors:** Pasquale F Innominato, Sandra Komarzynski, Ali Mohammad-Djafari, Alexandre Arbaud, Ayhan Ulusakarya, Mohamed Bouchahda, Mazen Haydar, Rachel Bossevot-Desmaris, Virginie Plessis, Magali Mocquery, Davina Bouchoucha, Mehran Afshar, Jacques Beau, Abdoulaye Karaboué, Jean-François Morère, Joanna Fursse, Jordi Rovira Simon, Francis Levi

**Affiliations:** ^1^Cancer Chronotherapy UnitCancer Research CentreWarwick Medical SchoolCoventryUnited Kingdom; ^2^Department of OncologyQueen Elizabeth HospitalBirmingham National Health Service Foundation TrustBirminghamUnited Kingdom; ^3^French National Institute for Health and Medical Research (INSERM)Unit 935VillejuifFrance; ^4^Laboratory of Signals and Systems (L2S)Unit 8506Gif-sur-YvetteFrance; ^5^Public Hospitals of Paris (AP-HP), Chronotherapy UnitDepartment of Medical OncologyPaul Brousse HospitalVillejuifFrance; ^6^Ramsay Générale de SantéMousseau ClinicsEvryFrance; ^7^St Georges HospitalNational Health Service Foundation TrustLondonUnited Kingdom; ^8^AK-SCIENCE, Research and Therapeutic InnovationVitry-sur-SeineFrance; ^9^Faculty of MedicineParis South UniversityLe Kremlin-BicêtreFrance; ^10^Chorleywood Health CentreChorleywoodUnited Kingdom; ^11^Telefónica Research and DevelopmentBarcelonaSpain

**Keywords:** domomedicine, chronotherapy, actigraphy, MDASI, telemonitoring

## Abstract

**Background:**

Telehealth solutions can improve the safety of ambulatory chemotherapy, contributing to the maintenance of patients at their home, hence improving their well-being, all the while reducing health care costs. There is, however, need for a practicable multilevel monitoring solution, encompassing relevant outputs involved in the pathophysiology of chemotherapy-induced toxicity. Domomedicine embraces the delivery of complex care and medical procedures at the patient’s home based on modern technologies, and thus it offers an integrated approach for increasing the safety of cancer patients on chemotherapy.

**Objective:**

The objective was to evaluate patient compliance and clinical relevance of a novel integrated multiparametric telemonitoring domomedicine platform in cancer patients receiving multidrug chemotherapy at home.

**Methods:**

Self-measured body weight, self-rated symptoms using the 19-item MD Anderson Symptom Inventory (MDASI), and circadian rest-activity rhythm recording with a wrist accelerometer (actigraph) were transmitted daily by patients to a server via the Internet, using a dedicated platform installed at home. Daily body weight changes, individual MDASI scores, and relative percentage of activity in-bed versus out-of-bed (I<O) were computed. Chemotherapy was administered according to the patient medical condition. Compliance was evaluated according to the proportions of (1) patient-days with all data available (full) and (2) patient-days with at least one parameter available (minimal). Acceptability was assessed using the Whole Systems Demonstrator Service User Technology Acceptability Questionnaire. Linear discriminant analysis was used to identify the combination of parameters associated with subsequent unplanned hospitalization.

**Results:**

A total of 31 patients (males: 55% [17/31]; World Health Organization Performance Status=0: 29% (9/31); age range: 35-91 years) participated for a median of 58 days (38-313). They received a total of 102 chemotherapy courses (64.7% as outpatients). Overall full compliance was 59.7% (522/874), with at least one data available for 830/874 patient-days (95.0%), during the 30-day per-protocol span. Missing data rates were similar for each parameter. Patients were altogether satisfied with the use of the platform. Ten toxicity-related hospitalizations occurred in 6 patients. The combination of weighted circadian function (actigraphy parameter I<O), body weight change, and MDASI scores predicted for ensuing emergency hospitalization within 3 days, with an accuracy of 94%.

**Conclusions:**

Multidimensional daily telemonitoring of body weight, circadian rest-activity rhythm, and patient-reported symptoms was feasible, satisfactory, and clinically relevant in patients on chemotherapy. This domomedicine platform constitutes a unique tool for the further development of safe home-based chemotherapy administration.

## Introduction

Incremental advances in the treatment of cancer are responsible for its progressive transformation into a chronic condition, with a similar level of impact on the individual patients’ quality of life, health, and well-being as other chronic diseases [1-3]. However, health care providers have not kept pace in developing the design of their management packages for cancer patients to fit the model of care for chronic diseases [1,2]. An important aspect of the chronic diseases model of care is delivery of treatment and review of symptoms assessed in the patient’s home and usual environment [4-8]. To best achieve this goal in cancer patients receiving often complex and toxic multidrug chemotherapy, it is important that the care model integrates the need for adequate safety [4,9,10]. Thus, an oftentimes complex compendium of procedures given to home-dwelling patients might be required [11]. Consequently, current practice usually involves chemotherapy administration at least partly within the hospital setting either as inpatient or outpatient, which can affect patient’s quality of life and increase the financial burden on patients and national health systems [5,9]. However, the integrated home care and support proposed with the domomedicine approach could offer an alternative to the present care system [12]. Domomedicine is defined as all procedures and care, sometimes complex, given at the patient’s home or in his or her social and professional activities, at least comparable in quantity and quality to those delivered in hospital, based on modern technologies, and it aims at promoting medical progress [12].

Improvements in treatment safety and patient well-being have been the mainstay for the development of the delivery methods that enable tailoring chemotherapy delivery according to circadian clocks in cancer patients. Toward this goal, cancer chronotherapy protocols use dedicated multichannel programmable-in-time pumps [13,14]. Thus, chronotherapy usually involves the chronomodulated delivery of chemotherapeutic agents according to circadian rhythms in nonhospitalized patients [13,15]. Doublet and triplet chronomodulated regimens have been safely administered at the patient’s home, resulting in improved tolerability and efficacy [16-19].

Recent progress in information and communication technologies can provide health care professionals with continuous data flow on symptoms, quality of life, toxicity, behavior, and circadian function from remote patients remaining within their own environment [7]. Toward this goal, we integrated daily telemonitoring of multidimensional objective and subjective parameters into a dedicated electronic home-based platform connected to the oncology department via a central server. We assessed the feasibility of such an approach and its acceptability in the clinical setting of advanced patients receiving multidrug chronomodulated chemotherapy at home. The clinical relevance of this domomedicine patient-centered system was further evaluated to provide a first estimate of its ability to predict unplanned emergency hospitalizations. The study was conducted within the framework of the inCASA European project (ICT-PSP). Its overall goal was the development of citizen-centric technologies and a service network to improve the health condition and daily life of patients suffering from a chronic disease, thus minimizing hospitalizations.

## Methods

### Patients and Setting

Patients aged more than 18 years with any cancer type requiring chemotherapy for at least one month were screened for the study at the Chronotherapy Clinics in the Medical Oncology Department of Paul Brousse Hospital in Villejuif, France. Eligibility further required the availability of an Internet connection at home and signed written informed consent. The inCASA electronic platform and the related equipment were installed at the home of each registered patient for a minimum of 30 days. The platform was connected to the Internet Protocol network. Each patient was instructed on how to use the platform for the daily transfer of biomedical data, and was given a form with telephone contacts information for technical or health-related issues.

While on study, patients could receive either conventional chemotherapy or chronotherapy according to medical decision. World Health Organization (WHO) Performance Status (PS) score was estimated for all patients before each treatment to support medical decision. WHO PS is a 0-5 score (0 indicating perfect health and 5, death) used to quantify cancer patients’ well-being and functional status [20]. All conventional treatments were administered in hospitalization or in outpatient clinics. Chronotherapy was delivered using a multichannel programmable pump (Melodie, Domocare, Montmirail, France) at home or during hospitalization, according to patient preference or medical decision.

The study was approved by the local institutional review board and conducted according to the Declaration of Helsinki [21]. Each patient signed a written informed consent form.

### Technical Equipment

**Figure 1:**
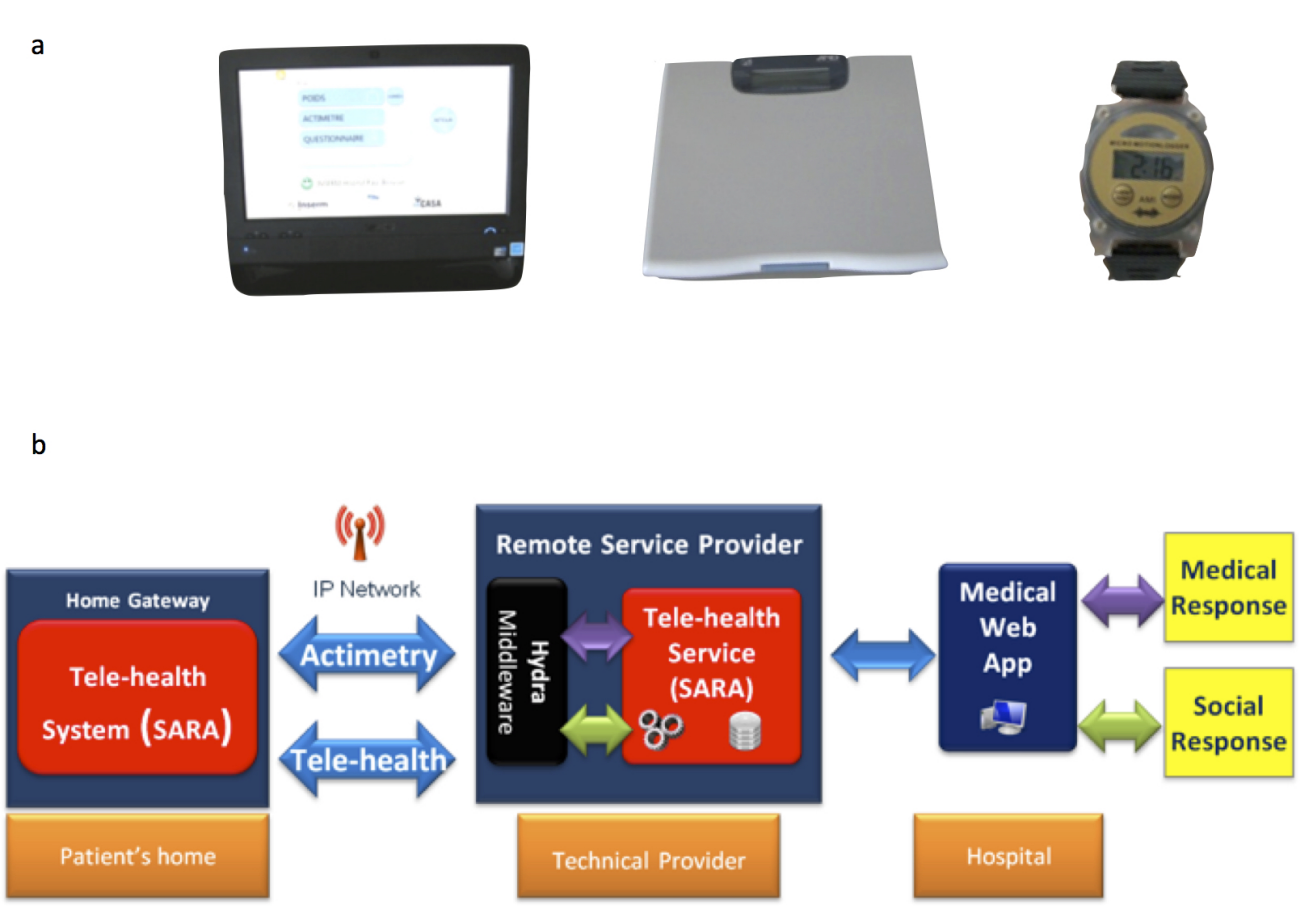
inCASA platform elements: (a) patient’s equipment (computer, scale, and actigraph); (b) information system technical architecture.

The inCASA platform was composed of (1) a touch screen computer (ASUS Eeetop ET1611, ASUSTEK, Taipei, Taiwan) equipped with the SARA software (Telefonica Investigacion y Desarrollo SA, Granada, Spain); (2) a body weight scale (UC321-PBT, A&D Medical, San Jose, CA, USA), which was connected to the computer via Bluetooth through the SARA application; and (3) a wrist-watch accelerometer (actigraph) (Micro MotionLogger, Ambulatory Monitoring Inc, Ardsley, NY, USA), whose collected data on wrist accelerations (per 1-min epoch) were transmitted via an infrared USB dongle connected to the computer (Figure 1a).

The SARA software included an electronic version of the MD Anderson Symptom Inventory (MDASI) questionnaire for self-assessment of 13 frequent core symptoms and 6 items assessing interference with activities of daily living. Daily self-rated MDASI items, self-measured body weight, and 24-h rest-activity records were automatically transmitted to a server through the Internet via the SARA software and the LinkSmart Middleware (Figure 1b).

### Study Design and Remote Monitoring Protocol

**Figure 2:**
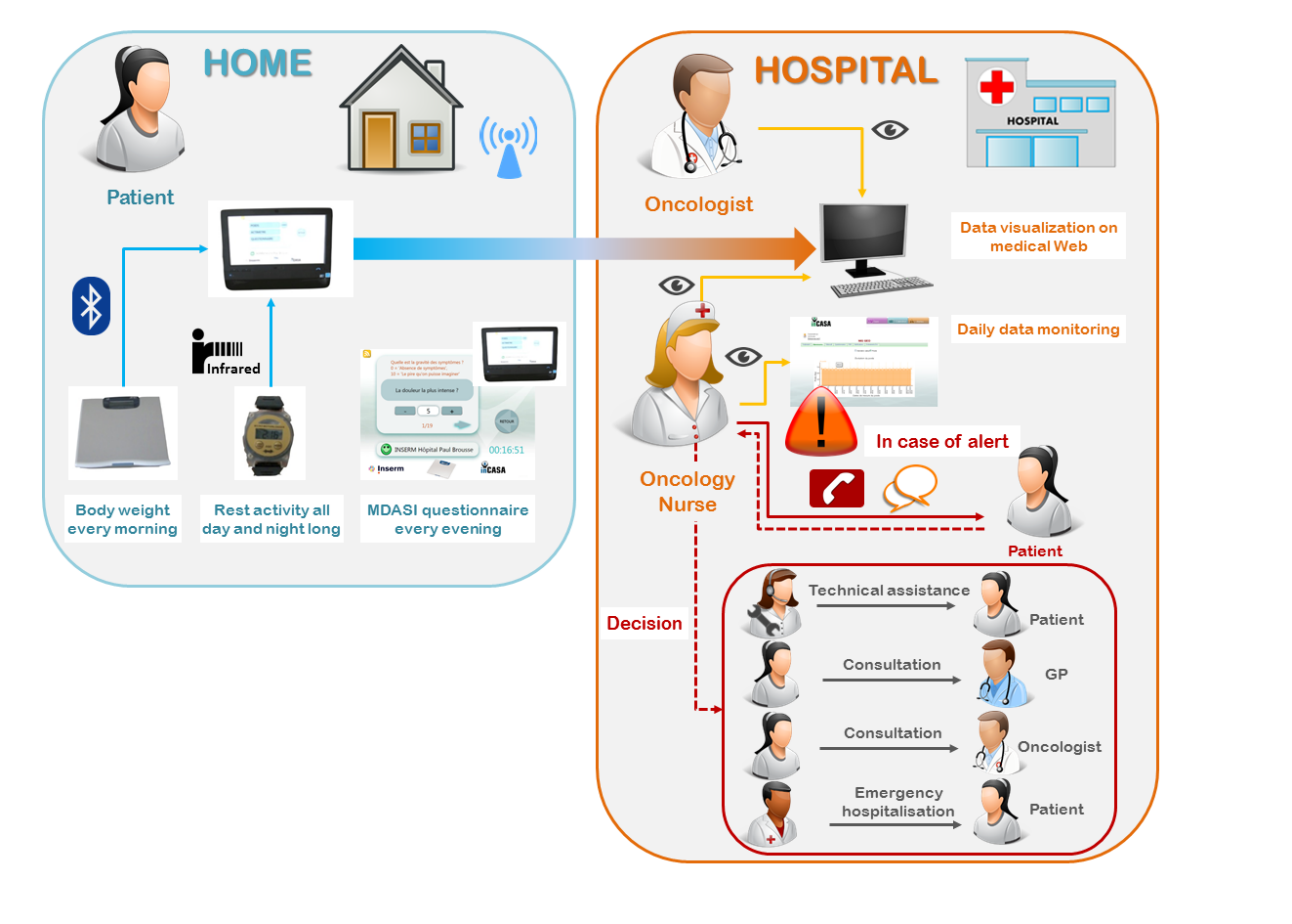
Outline of study design and remote monitoring procedures. Source of icons: pixabay.

Patients were instructed to weigh themselves each morning, fill out the on-screen MDASI questionnaire each evening, continuously wear the wrist actigraph, and download the data in the evening, before or after completing the questionnaire. All the data were then transmitted daily via the Internet to the secured central server. They could be securely accessed anytime by the oncology nursing or medical staff with a dedicated graphical display (Figure 2). The per-protocol recommended study duration was 30 days for each patient. Patients were asked to extend the duration of their participation to the study for further 30 days or more, depending upon their wishes and platform availability.

Patients were asked to contact the hospital or their general practitioner (GP) as they would have normally done in case of a health concern. Nonetheless, in case of a lack of data transmission for more than 24 hours, high symptom severity, quick body weight loss, or apparent deterioration of the circadian activity pattern, the oncology nurse usually phoned the patient and organized any appropriate intervention. This could involve telephone reassurance, a home visit by a technician or a nurse, a patient visit to the GP or to the oncologist, or an emergency visit at the outpatient clinics or in hospitalization (Figure 2).

### Platform Use Compliance

The overall compliance to the platform was assessed by calculating longitudinal individual patient-day reporting rates. These were defined as the total number of days in which data were obtained divided by the duration of the period during which the platform was available for use for each participant. These rates were calculated for each parameter (body weight, MDASI, and actigraphy) separately, for at least one parameter per patient-day, and for all 3 parameters together. Furthermore, given the 3-day time frame for emergency hospitalization prediction, we also calculated the percentage of patients with a full set of data (actigraphy, body weight, and MDASI) available at least once during a sliding window of 3 days. Longitudinal analysis was performed during the initial 30 days (primary endpoint), and for the following 30 days (on-study days 31–60), whenever applicable.

The study did not include specific and structured questioning about the reasons for missing data; however, during routine consultations, the medical oncologists (PI, AU, MB, MH, JFM, and FL) were encouraged to offhand discuss the noncompliance issue with the patients.

### Platform Evaluation

Participants rated their perception and satisfaction regarding the service delivered using the Whole Systems Demonstrator Service User Technology Acceptability Questionnaire (SUTAQ) [22] at study completion.

Unstructured, narrative interviews of the hospital nurses involved (RBD, VP, and MM) were performed by the main study investigators (AA, PI, and FL) to evaluate their global perception of the clinical relevance of the platform and acknowledge specific issues.

### Collected Data Analysis

The daily percentage of body weight change was calculated with reference to baseline values obtained over at least three days before the initial course of on-study chemotherapy. The 19 MDASI item scores were used without any predefined threshold. The rest-activity pattern was analyzed using the Action 4 software (Ambulatory Monitoring Inc). The dichotomy index I<O was selected as being the most clinically relevant in cancer patients, according to prior work [23-31]. I<O was computed as the percentage of activity epochs when in-bed, whose values were lower than the median level of activity when out-of-bed [32]. A normal dichotomy index is one approaching 100%, indicating restful sleep in bed at night and regular and lively activity during the day, out of bed. Values of I<O in healthy controls are rarely <98% [32,33]. Here, I<O was calculated over 72 h, with 3-day sliding windows, throughout the whole time series in each patient.

Descriptive analyses were performed for the overall distribution and individual longitudinal patterns of body weight change, of the 19 MDASI items separately, and of I<O.

### Emergency Hospitalization Prediction

Time series were analyzed using Matlab (The Mathworks, Natick, MA, USA), and ranked for relevance regarding prediction of emergency hospitalizations. First, handling missing data in each of the 21 time series (19 MDASI items, body weight loss, and I<O) from each patient were interpolated according to their localization: missing data localized at the beginning (or at the end) of the time series were assumed as having the value of the first (or the last) measured value, respectively; a linear interpolation was used to compute missing data within two measured data segments. We initially tested several interpolation approaches (bi-cubic, harmonic, likelihood, and principal component analysis-based) for our sensitivity analyses, but the final results were roughly similar to those using the simple linear method (data not shown). Second, we calculated the dynamic patterns of change over time by subtracting the parameter value of each day from the average of the same parameter on the three previous days. Finally, linear discriminant analysis (LDA) was used on these computed values to determine the combination of parameters whose 3-day dynamic patterns best predicted for an unplanned hospitalization (target event). The predefined time frame for prediction was set at the 3 days preceding each emergency hospitalization event.

## Results

### Study Patients’ Cohort

**Figure 3:**
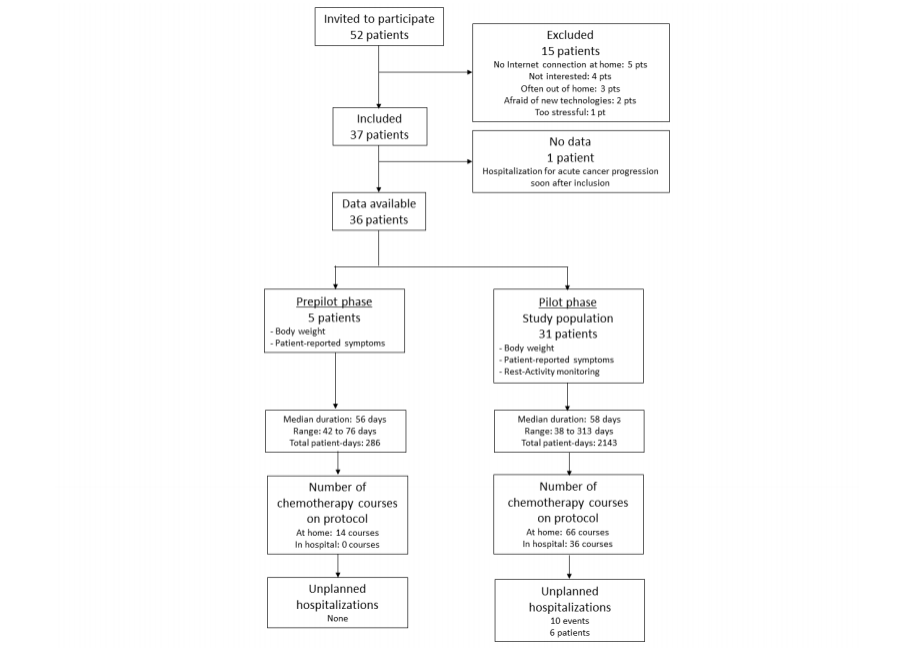
Study flowchart (Consort diagram).

A total of 52 patients were screened as potentially eligible from October 2011 to August 2013 (Figure 3). Eight were not registered for technical reasons, and 7 declined participating. One patient was repeatedly hospitalized for prolonged spans because of acute cancer progression just after inclusion and could not provide any data. Five patients participated in the prepilot phase. The results reported here regard the 31 patients included in the pilot phase.

Patients aged 35-91 years (median: 61 years) participated in the study for a median duration of 58 days (range: 38-313 days). Most of them were treated for colorectal, pancreatic, or breast cancer (Table 1). The majority of patients had undergone prior surgery and received prior chemotherapy. A total of 102 chemotherapy courses were administered to the 31 patients while on-study. Six patients (19%) received 20 courses of conventional chemotherapy at the hospital (20% of courses). The remaining 25 patients (81%) were treated with 82 courses of chronomodulated chemotherapy, of which 66 (80%) were administered at home.

**Table 1:** Clinical features of the study population.

Patient characteristics		n (%)
**Gender**		
	Male	17 (55)
	Female	14 (45)
**Age (years)**		
	35-45	4 (13)
	45-55	4 (13)
	55-65	11(36)
	65-75	5 (16)
	75-85	5 (16)
	85-95	2 (6)
	Median (range)	61 (35-91)
**World Health Organization performance status**		
	0	9 (29)
	1	11(36)
	2	2 (6)
	3	2 (6)
	Not available	7 (23)
**Primary tumor site**		
	Colon	8 (26)
	Rectum	4 (13)
	Pancreas	9 (29)
	Breast	5 (16)
	Prostate	2 (7)
	Lung	1(3)
	Liver	1(3)
	Ovary	1(3)
**Number of metastatic sites**		
	0	7 (23)
	1	24 (77)
**Comorbidities**		
	None	24 (78)
	Diabetes	5 (16)
	Hepatitis B	1 (3)
	Chronic heart failure	1(3)
**Prior surgery for**		
	None	3 (10)
	Primary tumor	20 (64)
	Metastases	8 (26)
**Prior chemotherapy**		
	No prior chemotherapy	3 (9)
	Adjuvant only	11 (36)
	Metastatic only	1 (3)
	Both	16 (52)
**Number of prior chemotherapy protocols for metastatic disease**		
	None	13 (42)
	One	4 (13)
	Two or more	12 (39)
	Unknown	2 (6)
**Chronotherapy protocol while on inCASA**		
	Total number	25 (81)
	ChronoIFLO4^a^	13 (42)
	Other chrono triplets	2 (7)
	Other chrono doublets	10 (32)
**Conventional chemotherapy protocol while on inCASA**		
	Total number	6 (19)
	Triplet	1 (3)
	Doublet	2 (6)
	Monotherapy	3 (10)
**Protocol courses given on inCASA**		
	Total number	102 (100)
	At home	66 (65)
	At hospital	36 (35)
	Median number per patient (range)	3 (1-14)

^a^ChronoIFLO4 is the chronomodulated combination of irinotecan, oxaliplatin, 5-fluorouracil, and leucovorin [19].

### Study Compliance

**Figure 4:**
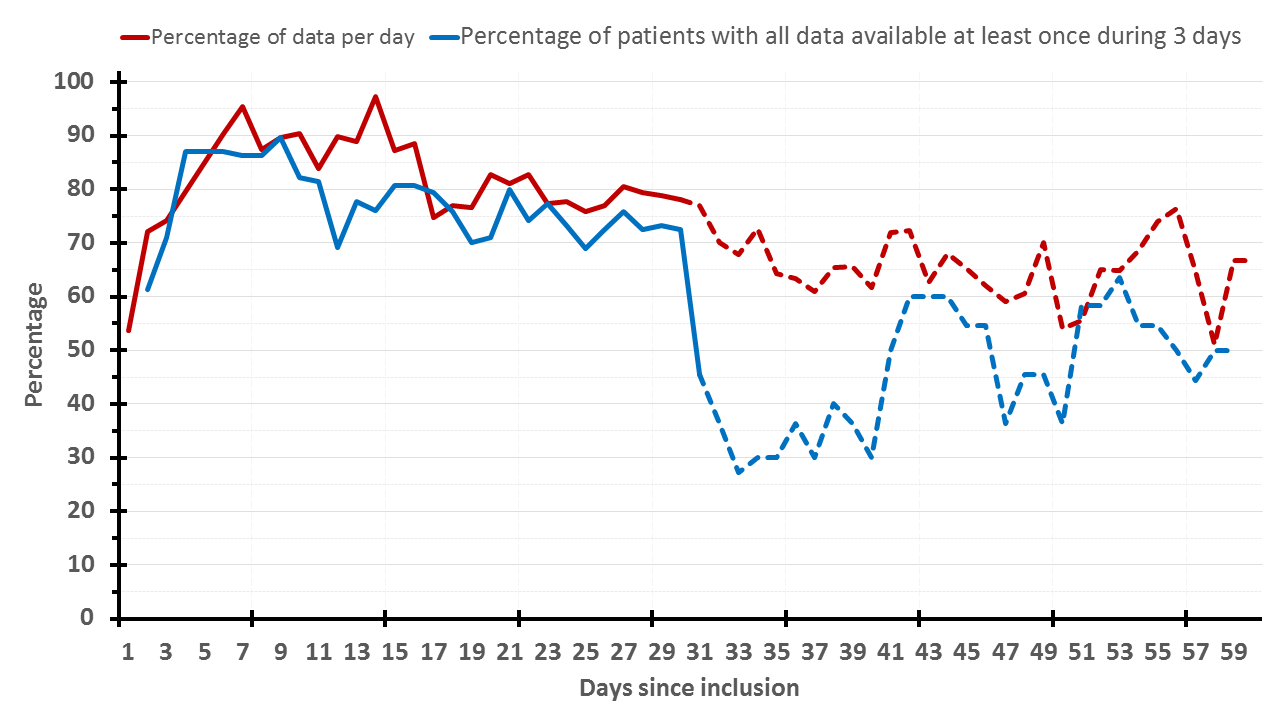
Long-term longitudinal compliance rates. The red curve shows the percentage of available data per day (MDASI counted as one), and the blue one, the percentage of patients with all data (actigraphy, body weight, and MDASI) available at least once during 3 days. The solid curves plot the initial 30 days (per-protocol period), and the dashed ones, the following 30 days (day 31-60). MDASI: MD Anderson Symptom Inventory.

During the initial 30-day period (per-protocol), patients provided complete daily data (body weight, MDASI, and actigraphy) for a total of 522 days out of the 874 theoretical patient-day data (930 total minus the 56 days of elective or emergency hospitalizations). Hence, overall full compliance was 59.7%. Individual general compliance for each parameter was as follows: 81.7% (714/874) for body weight, 78.1% (683/874) for MDASI, and 74.7% (653/874) for actigraphy. At least one parameter for each patient-day was available in 95.0% (830/874) cases. Moreover, at least one complete set of daily data for the 3 parameters was available at least once every 3 days in 77.2% of the cases. Altogether, compliance remained rather good and stable over the per-protocol 30-day span (Figure 4). However, in the longer term (days on study 31-60), data availability decreased for those patients who opted for continuing the study beyond the per-protocol time span (Figure 4). In particular, over this subsequent 30-day span, complete daily data were provided for 38.7% (264/683) patient-days. Respective figures were 64.4% (440/683) for body weight, 61.3% (419/683) for MDASI, 62.1% (424/683) for actigraphy, and 83.0% (567/683) for at least one of them. Finally, 55.1% of patient-days had at least one complete set of data at least once every 3 days, during days on study 31-60 (Figure 4).

Individual patient compliance (available out of theoretical data) ranged from 0% to 85.7%, with a median of 56.3%.

The most common reasons for missing data, outside planned or emergency hospitalizations (125 patient-days), were informally reported to be technical problems, out-of-home trips, and patient forgetting or feeling too sick.

### Platform Evaluation

Fifteen patients completed the SUTAQ questionnaire, which was offered at mid study course to 22 patients (results detailed in Table 2). The general satisfaction rate was 84%. The system was perceived to enhance care for 80% of the patients; 87% of the participants indicated that it did not interfere with their life or privacy. However, 67% of them considered that it could not be used as a substitution for the current health care. No patient in the study offhand recounted any major issues appreciated in the platform to the physicians, nurses, or technicians. Whenever specifically questioned, patients expressed generally positive comments on the platform, as a usable additional health care tool, in agreement with the subgroup completing SUTAQ.

All 3 surveyed hospital nurses involved in this study spontaneously reported the perception that the system globally improved the follow-up of the health condition of the patients, in comparison to the current standard procedure. They also acknowledged, nevertheless, that some technical problems interfered with their experience, especially when recurring in the same patient, and proposed that dedicated personnel ought to be allocated to such domomedicine task. Finally, the system usability was altogether recognized by the 3 nurses independently as operational for larger-scale deployment.

**Table 2:** Perception and satisfaction results^a^.

	SUTAQ^b^ items						
Outcomes	Enhanced care	Increased accessibility	Privacy and discomfort	Care personnel concerns	Kit as substitution	Satisfaction
Mean item score	3.8	3.0	4.2	3.6	3.1	4.2
General satisfaction with the item	77%	60%	83%	72%	62%	84%
Number of patients satisfied with the item	12	7	13	10	5	14
Percentage of patients satisfied with the item	80%	47%	87%	67%	33%	93%

^a^Responses to the SUTAQ questions were measured using a 5-point Likert scale.

^b^SUTAQ: Service User Technology Acceptability Questionnaire.

**Table 3:** Distribution of objective and subjective collected data during the study.

Parameters			Median	1st; 3rd quartiles	Range (min to max)
**Objective**								
	Body weight change (%)		–0.4	–2.0; 0.9	–10.5 to 6.0
	I<O (%)		98.3	96.6; 99.1	82.5 to 100
**Subjective**					
	**MDASI^a^ symptom items**							
		Pain	2	0; 4	0 to 10
		Fatigue	4	2; 5	0 to 10
		Nausea	0	0; 2	0 to 10
		Disturbed sleep	2	0; 4	0 to 9
		Distress	3	1; 5	0 to 10
		Shortness of breath	2	1; 5	0 to 9
		Problem with remembering things	1	0; 2	0 to 8
		Lack of appetite	2	0; 5	0 to 10
		Drowsiness	2	0; 4	0 to 8
		Dry mouth	1	0; 5	0 to 10
		Sadness	2	0; 5	0 to 9
		Vomiting	0	0; 0	0 to 10
		Numbness or tingling	1	0; 3	0 to 10
	**MDASI interference items**					
		General activity	4	2; 5	0 to 10
		Mood	2	0; 4	0 to 9
		Work	5	2; 6	0 to10
		Relations with others	2	0; 4	0 to 9
		Walking	3	1; 5	0 to 10
		Enjoyment of life	3	2; 5	0 to 10

^a^MDASI: MD Anderson Symptom Inventory.

### Descriptive Analysis of Collected Data

The quartiles and extreme values of body weight change, I<O, and individual MDASI items for all patients throughout the whole study span are shown in Table 3.

**Figure 5:**
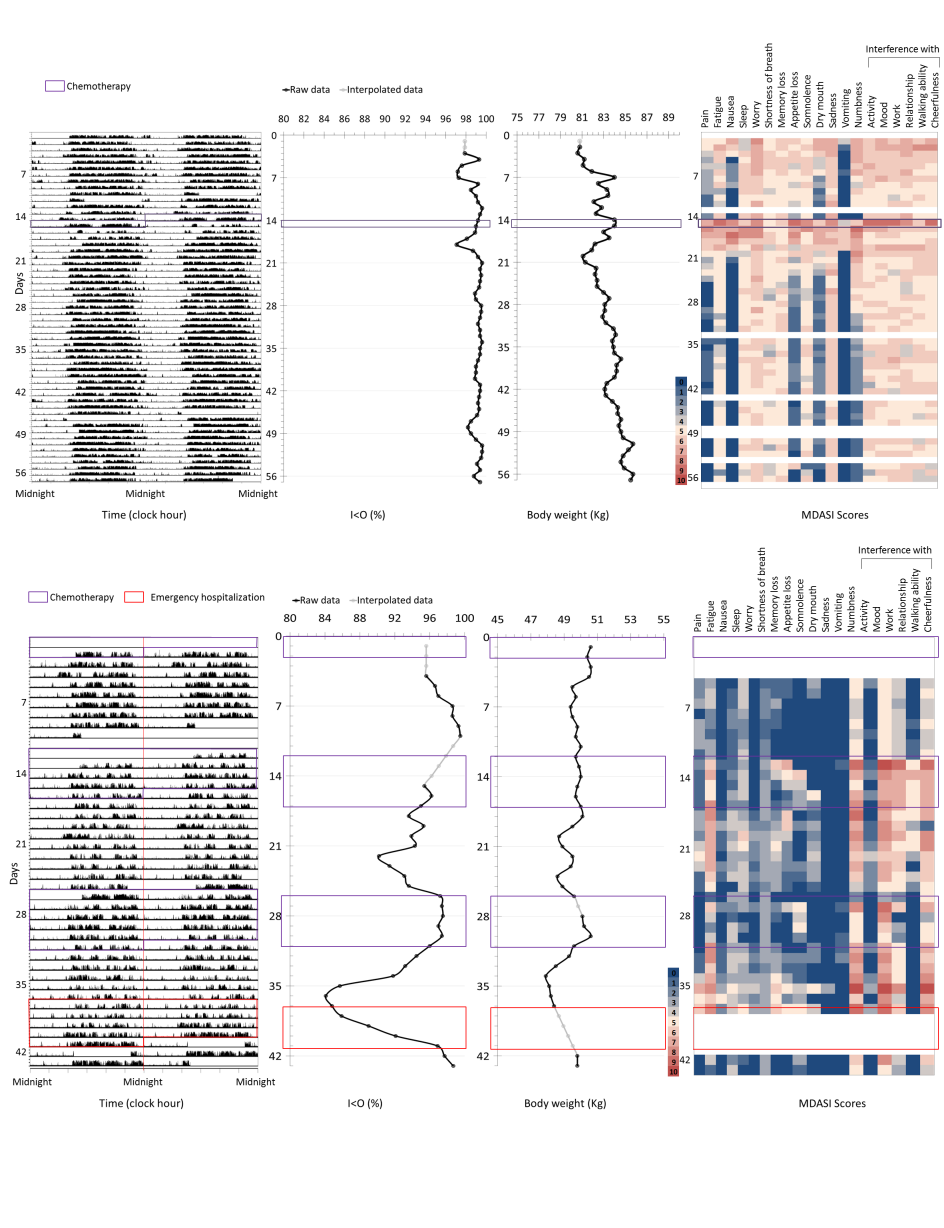
Representative examples of the multidimensional data available for 2 patients over 57 (top) and 44 (bottom) monitoring days, respectively. From left to right: first panels: actigraphy recording (midnight-centered double plot; Y-axis: activity counts per minute); second panels: corresponding daily I<O values; third panels: daily body weight change; fourth panels: daily MDASI items (heat map; white represents missing values, blue through yellow to red, increasing values from 0 to 10). Purple boxes represent the days during which chemotherapy was administered. In the bottom plot, the red box represents the duration of an emergency hospitalization. MDASI: MD Anderson Symptom Inventory.

The dynamics of rest-activity patterns, computed I<O values, body weight changes, and MDASI items scores are depicted for 2 representative patients over 57 and 44 days, respectively (Figure 5).

### Emergency Hospitalizations

An emergency hospitalization event occurred after 9.8% (10/102) chemotherapy courses in 6 patients (19%). Thus, 5 participants underwent a single unplanned hospitalization, and 1 patient was hospitalized 5 times. Table 4 details the most relevant characteristics of these hospitalization events. Appropriate symptomatic treatment was administered as indicated, with discharge at home in all cases.

**Table 4:** Clinical features of patients involved and events regarding unplanned hospitalizations.

Patient characteristics			n (%)
**Gender**			
	Male		2 (33)
	Female		4 (67)
Age in years, median (range)			60 (52-91)
**Chemotherapy protocols followed by unplanned hospitalization, n=10**			
	**Type of delivered chemotherapy**		
		Chronotherapy	9 (90)
		Conventional chemotherapy	1 (10)
	**Location of treatment delivery**		
		At home	5 (50)
	
		At hospital	5 (50)
	
**Hospitalizations, n=10**			
	**Number per patient**		
		Only one hospitalization	5 (83)
	
		More than one hospitalization	1 (17)
	
		Hospital stay (days), median duration (range)	6 (2-9)
**Causes**			
		Gastrointestinal symptoms with general physical deterioration	5 (50)
		Febrile neutropenia	3 (30)
		Sepsis	1 (10)
		Asthenia with poor general condition	1 (10)

### Early Warning Signals Predicting Emergency Hospitalizations

**Figure 6:**
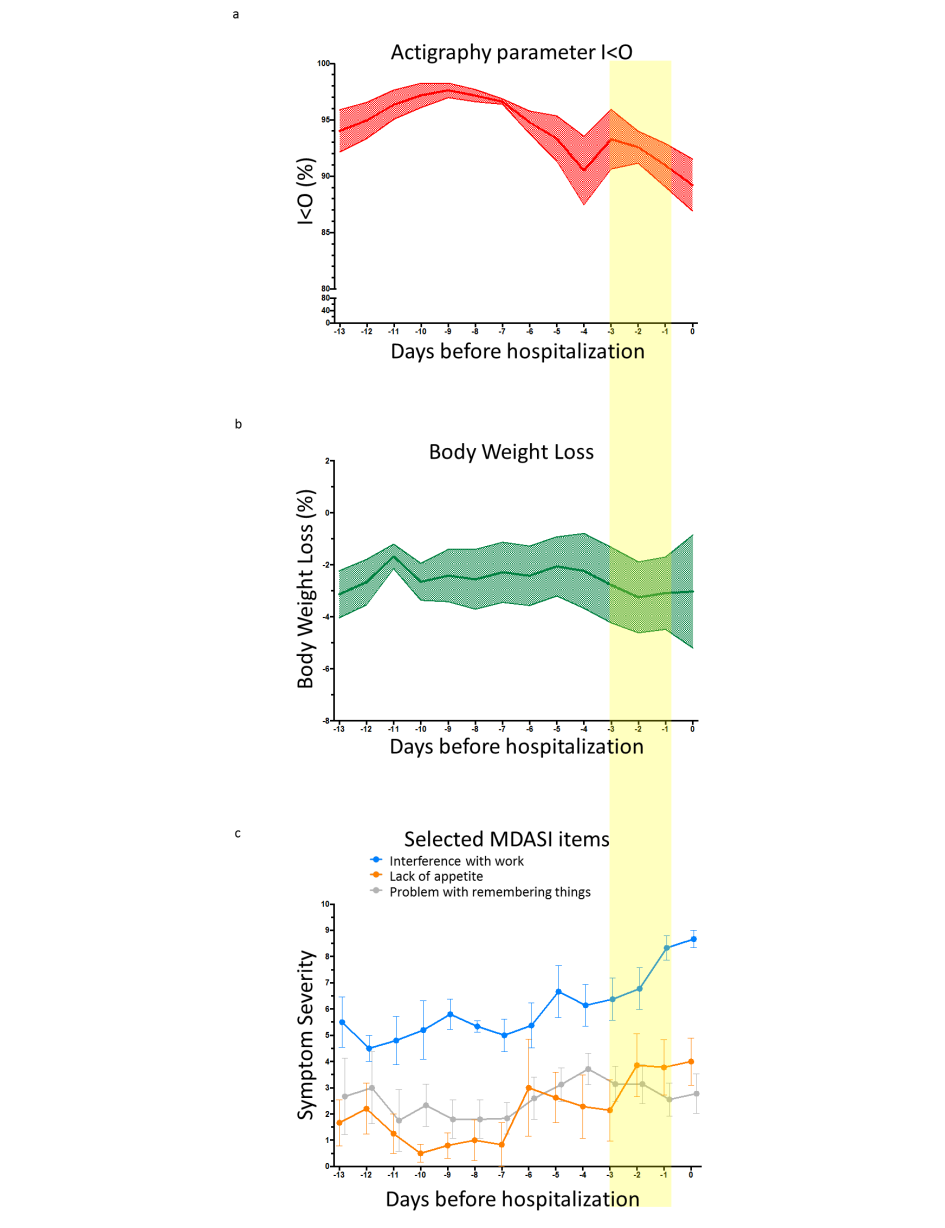
(a) Average (and standard error of the mean) daily I<O values, (b) body weight change, (c) and 3 selected MDASI items in the 2 weeks preceding unplanned hospitalizations (n=10). The yellow bar highlights the 3 days preceding the emergency hospitalization, used for the predictive analysis (LDA). MDASI: MD Anderson Symptom Inventory, LDA: linear discriminant analysis.

A global decrease in average daily rest-activity I<O values was observed over the 2 weeks preceding an unplanned hospitalization (Figure 6a). No such trend was obvious for body weight changes (Figure 6b). Some patient-reported symptoms, such as interference with work or lack of appetite, appeared to worsen on average before an unplanned admission, while others, such as problem with remembering things, did not display congruous changes over the same time span (Figure 6c).

LDA identified the model with the relative weights for the dynamic patterns in circadian rest-activity I<O parameter, body weight change, and MDASI scores, which best predicted for a subsequent emergency hospitalization during the following 3 days (Table 5). Testing the model on the whole dataset (initial 30 days for learning and additional 30 days for validation) yielded a sensitivity of 55.5%, a specificity of 94.6%, a positive predictive value of 12.7%, and a negative predictive value of 99.3% (Table 6). Hence, global accuracy was 94.0%. Sensitivity analyses confirmed the results of the main predictive model, with the highest loading weight assigned to I<O and a cluster of MDASI items connected to interference with relations, daily activities, and appetite.

**Table 5:** Coefficients of all the 21 items (ranked) of the final predictive linear discriminant analysis (LDA) model.

Parameter	Coefficient
Shortness of breath	–0.945
I<O	0.772
Relations with others	0.634
Work	–0.492
Disturbed sleep	0.473
Drowsiness	–0.466
Sadness	0.435
Distress	–0.351
Problem with remembering things	–0.335
Vomiting	0.283
Numbness or tingling	0.221
Mood	0.198
General activity	0.153
Nausea	–0.136
Enjoyment of life	–0.128
Dry mouth	0.097
Walking	0.089
Lack of appetite	–0.089
Pain	0.065
Body weight change	–0.025
Fatigue	–0.009

**Table 6:** Confusion matrix regarding prediction of unplanned hospitalization.

Patient/day	Actual event		
Predicted	Yes	No
Yes	10	69
No	8	1203

## Discussion

### Main Results

Continuous telemonitoring of circadian rest-activity rhythm, jointly with daily body weight and self-reported symptoms, was implemented for the first time in the home of patients receiving chemotherapy for advanced cancer. The inCASA domomedicine platform was found to be adequate for such purpose, since it was well accepted by the patients and provided an unprecedented amount of multidimensional data over prolonged time spans. Furthermore, the integration of the subjective and objective data translated into accurate information able to predict subsequent emergency hospitalization (Table 6). Thus, such novel telehealth system carries out a number of potential clinical applications.

### Comparison With Prior Studies

The inCASA platform was home located and Web-based, but it did not require to log-in, in contrast to other telehealth studies [5,34-36]. The multiparameter monitoring performed here was not limited to patient-reported outcomes, as it is usually the case [5,34,35,37]. In contrast, teletransmitted parameters also included objectively measured body weight and wrist activity pattern (Figure 2). This required technical innovations for minimizing patient discomfort and maximizing compliance. Both parameters were chosen on the basis of previous results relating altered circadian rest-activity rhythm to poor outcomes [23,25,27], body weight loss to circadian disruption [24], and both parameters to poor survival outcome on chronotherapy [38]. The proper tracking of the dynamic changes in these parameters entailed the need for daily measurements, whereas other authors have proposed evaluations of symptoms and quality of life once a week or at each clinic visit [4,5,34,35,39,40]. With an overall 59.7% per-protocol compliance rate, and with 95% of the days having at least one data, our study demonstrates the feasibility of cancer patients’ empowerment for gathering and teletransmitting both subjective and objective health-related data. Moreover, the compliance appeared altogether stable over time during the per-protocol span (Figure 4), inferring possible long-term use. However, the sustainability of such platform in daily clinical practice cannot be definitely foretold with this study, as it did not include cost-effectiveness analysis or payment stakeholders’ involvement.

### Limitations

Although the platform workflow was well accepted by patients altogether, some technological aspects could be refined to increase both comfort and convenience, thus further improving compliance rates in view of a prolonged use. For example, circadian rest-activity rhythm data could be automatically and seamlessly transmitted to the home-based platform, and thereupon to the central server, resulting in less end-user manipulations. Additionally, hospital nurses indicated the positive impact of timely technical support for the implementation of this patient-centered approach, confirming previous report [41]. On some occasions, patients were unable to provide data due to traveling away from home, suggesting the relevance of lightweight mobile systems for further developments. Thus, a handheld computer was used to record self-reported and objective measures in oncologic outpatients with satisfactory acceptance and reliability [39]. Similarly, smartphone-based apps have been successfully tested for frequent symptom evaluation and toxicity management in patients on chemotherapy [9,42]. Such mobile technology could further enhance long-term compliance, as suggested [35,37]. However, certain features of the solution will need to be modified, including the addition of a log-in component for authentication on the main mobile device, required to be connected to the Web via secure wireless network for data transfer protocol, and the possibility of manually adding body weight. Nonetheless, the wearable device, the interface of the main technical equipment, the server-based system, and the remote monitoring procedures will allegedly not require significant modifications.

### Perspectives

Notwithstanding these amendable technical issues, this study identified, for the first time, a combination of subjective and objective parameters whose dynamics predicted the occurrence of emergency hospitalization within 3 days from the event with an accuracy of 94%. Thus, the integrated multiparametric assessment, including body weight and circadian rest-activity rhythm jointly with subjectively rated symptoms, provided a novel framework for the early detection of severe adverse events that will require hospital admission within 3 days. These data allow foreseeing the timely triggering of proactive interventions to improve the safety of treatment administration at home while potentially reducing the financial burden on health care providers [43,44]. Indeed, less frequent emergency room visits were described from adequate nurse-initiated response during routine cancer care involving weekly remote monitoring of self-reported symptoms [36].

This pilot study was mainly observational; hence, no predefined decisional pathway or procedures were implemented according to telemonitored data. Notwithstanding, common sense and prudence led the investigators to off-protocol contact patients in case of missing data, mainly for reasons not requiring medical attention, or, less often, in case of parameter deterioration (Figure 2). In this study we could not quantify the benefit to the patients related to early interventions prompted by observed alterations in I<O, body weight change, or MDASI items, but it was probably realistic to assume that, in some cases, contacting the patient and eliciting a rapid and opportune medical care response could have avoided more severe outcomes, even if these procedures were informally executed (Figure 2).

Such hypothesis of patient benefit from a telemonitoring-guided proactive intervention is being tested within a multicenter domomedicine French study, using a second-generation platform (PiCADo) [12]. The study involves patients receiving multidrug chronotherapy at home, using the acquired expertise and the a posteriori predictive model derived from the current study. Moreover, the forecasting analysis applied in the current study provides an evolving methodological framework, whose prediction ability improves through learning based on data enrichment stemming from forthcoming studies.

Finally, besides low I<O that defines altered circadian rhythms, the MDASI items most strongly associated with subsequent unplanned hospitalization have been linked to circadian disruption (fatigue, appetite loss, and poor physical, social, and role functioning) [23,24,26,27,45]. Therefore, these findings bolster the clinical relevance and warrant the implementation of circadian rhythms monitoring in medical oncology [15].

### Conclusions

The inCASA solution allowed monitoring not only of patient-reported symptoms, but also of circadian rest-activity patterns and of body weight in cancer patients on chemotherapy, while they were at home. These unique and novel data provided useful information to health care and social care professionals for the follow-up of patient’s well-being. The ultimate paramount benefit of this approach is the increased safety of chemotherapy administration at home. In our experience, this multidimensional telemonitoring represented an effective, accurate, and refined tool for identifying patients at risk for emergency hospitalization, allowing in the future the development and timely triggering of preemptive, coordinated, and befitting interventions to prevent such unplanned admissions, within a domomedicine approach.

## Abbreviations

I<O: dichotomy index
LDA: linear discriminant analysis
MDASI: MD Anderson Symptom Inventory
PS: performance status
SUTAQ: Service User Technology Acceptability Questionnaire
WHO: World Health Organization
